# HOXC9 characterizes a suppressive tumor immune microenvironment and integration with multiple immune biomarkers predicts response to PD-1 blockade plus chemotherapy in lung adenocarcinoma

**DOI:** 10.18632/aging.205637

**Published:** 2024-03-05

**Authors:** Liang Liu, Zhenshan Zhang, Chenxue Jiang, Yaoyao Zhu, Ruiqin Han, Leilei Wu, Yaping Xu

**Affiliations:** 1 Department of Radiation Oncology, Shanghai Pulmonary Hospital, School of Medicine, Tongji University, Shanghai 200433, China; 2 Department of Radiation Oncology, Shanghai Proton and Heavy Ion Center, Fudan University Cancer Hospital, Shanghai 201315, China; 3 State Key Laboratory of Common Mechanism Research for Major Disease, Institute of Basic Medical Sciences, Chinese Academy of Medical Sciences and Peking Union Medical College, Beijing 100191, China

**Keywords:** LUAD, HOXC9, immunotherapy, TME, biomarker

## Abstract

Background: The quest for dependable biomarkers to predict responses to immune checkpoint inhibitors (ICIs) combined with chemotherapy in advanced non-small cell lung cancer remains unfulfilled. HOXC9, known for its role in oncogenesis and creating a suppressive tumor microenvironment (TME), shows promise in enhancing predictive precision when included as a TME biomarker. This study explores the predictive significance of HOXC9 for ICI plus chemotherapy efficacy in lung adenocarcinoma (LUAD).

Methods: Following the bioinformatic findings, assays were performed to ascertain the effects of Hoxc9 on oncogenesis and response to programmed death 1 (PD-1) blockade. Furthermore, a cohort of LUAD patients were prospectively enrolled to receive anti-PD-1 plus chemotherapy. Based on the expression levels, baseline characteristics, and clinical outcomes, the predictive potential of HOXC9, PD-L1, CD4, CD8, CD68, and FOXP3 was integrally analyzed. HOXC9 not only mediated oncogenesis, but also corelated with suppressive TME. CMT167 and LLC cell lines unveiled the impacts of Hoxc9 on proliferation, invasion, and migration. Subsequently, tumor-bearing murine models were established to validate the inverse relationship between Hoxc9 expression and effective CD8+ T cells.

Results: Inhibition of Hoxc9 significantly curtailed tumor growth (P<0.05), independent of PD-1 blockade. In patient studies, while individual markers fell short in prognosticating survival, a notable elevation in CD8-positive expression was observed in responders (P=0.042). Yet, the amalgamation of HOXC9 with other markers provided a more distinct differentiation between responders and non-responders. Notably, patients displaying PD-L1+/HOXC9- and CD8+/HOXC9- phenotypes exhibited significantly prolonged progression-free survival.

Conclusions: The expression of HOXC9 may serve as a biomarker to amplifying predictive efficacy for ICIs plus chemotherapy, which is also a viable oncogene and therapeutic target for immunotherapy in LUAD.

## INTRODUCTION

Lung cancer is the leading cause of cancer death worldwide, with an estimated 1.8 million deaths in one year [1]. Non-small cell lung cancer (NSCLC) constitutes the predominant subset, accounting for 80-85% of cases, encompassing lung adenocarcinoma (LUAD) and squamous cell carcinoma (LUSC) [2, 3]. Over the past decade, the advance of immune checkpoint inhibitors (ICIs) has revolutionized the therapeutic landscape of lung cancer, especially targeting programmed death 1 (PD-1) and programmed death ligand 1 (PD-L1) [4]. In the realm of locally advanced or metastatic NSCLC, the combination of PD-1 [5, 6] or PD-L1 [7] blockade plus chemotherapy has demonstrated marked enhancements in both the progression-free survival (PFS) and overall survival (OS), compared with chemotherapy alone in the first-line setting, irrespective of the PD-L1 percentage. Despite the emergence as a core pillar of NSCLC treatment, nearly half of them could not benefit from this regimen, exhibiting objective response rates (ORR) spanning 48% to 64% [4]. Notably, a significant proportion eventually develops resistance to ICIs post initial response [3]. Hence, there is an urgent need to identify reliable predictive biomarkers for this therapeutic regimen.

Presently, PD-L1 expression, tumor mutational burden (TMB) and microsatellite instability (MSI) serve as officially sanctioned predictive biomarkers guiding ICI monotherapy, yet their screening accuracy remains wanting [3, 8]. In the context of ICIs combined with chemotherapy, the predictive capabilities of PD-L1 expression and TMB have not been conclusively established [9]. The intricate interplay between tumor cells and the tumor microenvironment (TME) underscores the crucial impact of immunotherapy, propelling TME to the forefront as a noteworthy source of predictive markers for immunotherapy and a potential therapeutic target [10, 11]. The TME encompasses an array of constituents, including diverse cancer cells, immune cells, vasculature, stromal elements, signaling molecules, and extracellular matrix proteins, manifesting a variety of markers, such as CD4+ helper T cells, CD8+ tumor-infiltrating lymphocytes (TILs), CD68+ tissue-associated macrophages, FOXP3+ regulatory T cells (Tregs), and interferon-gamma (IFN-γ) gene signature [3, 11, 12]. Given this complexity, there is increasing interest in leveraging distinct TME characteristics to develop more reliable predictive biomarkers for immunotherapy [11].

The Homeobox (HOX) gene family orchestrates the synthesis of transcriptional regulators pivotal in embryonic development, pivotal for cell morphogenesis and organ differentiation [13]. Homeobox C9 (HOXC9), a member of the HOX family [14], surfaces with anomalous expression across various cancer cell lines and tissues such as colorectal cancer [15], gastric cancer [16], and breast cancer [17]. For instance, in breast cancer cell lines, HOXC9 overexpression curtailed cell proliferation while bolstering invasive capabilities, akin to promoting a phenotypic shift from proliferation to invasiveness [17]. Recently, the role of HOXC9 in LUAD was explored to some extent. Bi et al. [18] elucidated its prognostic hazard in LUAD via hsa_circ_0020123/miR-495/HOXC9 axis. Similarly, Liu et al. [19] established a correlation between HOXC9 and the circRNA system in LUAD. Interestingly, they further found HOXC9 pertained to diminished infiltration of CD8+ T cells and dendritic cells, potentially contributing to antitumor immunity dysregulation and poorer outcomes for individuals with elevated HOXC9 expression, suggesting its prospective utility as a suppressive TME biomarker. However, the extent of its regulatory influence within the TME, as well as its predictive value for ICIs response and survival, necessitate further elucidation.

In the present study, we explored the regulatory role of HOXC9 on the TME both *in vivo* and *in vitro*. We then investigated the predictive role of HOXC9 in LUAD, comparing its efficacy with other markers in predicting ICIs responses and patient survival. Additionally, we explored the accuracy of different combinations, aiming to provide a more effective biomarker for personalized treatment.

## MATERIALS AND METHODS

### Gene expression analysis

We employed the online tools in the ‘Exploration’ mode of Tumor Immune Estimation Resource 2.0 (TIMER2) (http://timer.cistrome.org/) [20] to acquire diverse expression patterns of HOXC9 across various cancers and neighboring healthy tissues. The raw data underwent normalization through log2(TPM) (Transcripts Per Kilobase of exon model per Million mapped reads) conversion.

### Genetic alteration and promoter

The estimation of HOXC9 genetic changes was conducted using the web-based cBioPortal repository (https://www.cbioportal.org/) [21, 22]. This analysis relied on data from the TCGA pan-cancer atlas research database [23]. The mutation distribution of HOXC9 was graphed using the Catalogue of Somatic Mutations (COSMIC) (http://www.sanger.ac.uk/cosmic/) [24], a publicly available resource that provides details about somatic mutations found in human cancers.

### Survival prognosis analysis

Visualized through R’s survival and survminer packages, the Kaplan-Meier assessment of LUAD’s HOXC9, derived from the TCGA database alongside clinical data from our hospital, was conducted. The computed values encompassed the log-rank P-value, hazard ratio (HR), and 95% confidence intervals (CI).

### Cells and reagents

The CMT167 and Lewis lung cancer (LLC) cell lines derived from mice were procured from European Collection of Authenticated Cell Cultures (ECACC), and the American Type Culture Collection (ATCC). They were nurtured in DMEM medium (Gibco, Thermo Fisher Scientific, Inc., USA), supplemented with 10% fetal bovine serum (Thermo Fisher Scientific, Inc.) and 1% Penicillin-Streptomycin (Solarbio, Beijing, China). Incubation was carried out in a 37° C environment with 5% CO_2_ and 95% humidity. Following procurement, the cell lines underwent authentication through Short Tandem Repeat (STR) analysis, and routine screenings verified their negative status for mycoplasma contamination.

### Hoxc9 overexpression and knockdown construction

In order to create knockdown of Hoxc9 cells, two small interfering RNA (siRNA) molecules targeting Hoxc9 (siHoxc9-1 and siHoxc9-2), along with a control siRNA (siCtrl), were generated and synthesized by Shanghai GenePharma Co. The initial step involved seeding lung cancer cells into six-well plates, proceeding with transfection when cell density reached 80–90% confluence. Lipofectamine3000 reagent (Invitrogen; Thermo Fisher Scientific, Inc.) was employed to transfect CMT167 and LLC cells with siRNAs or siCtrl, following the manufacturer’s provided protocols. The antisense sequences corresponding to the three siRNAs were displayed as follows:

siHoxc9-1: 5′- CAGTCGTCTGTGGTCTATCACCCTT-3′; siHoxc9-2: 5′- AGTCGTCTGTGGTCTATCACCCTTA-3′; siCtrl: 5′-CAGCTGTGTTGGATCACTCCTCCTT-3′.

Immunoblotting and qPCR techniques were utilized to confirm gene expression levels. The sequence of siHoxc9-1 informed the creation of shRNA constructs. These constructs, along with lentivirus packaging vectors, were introduced into HEK-293T cells, facilitating the generation of lentiviral particles for either shRNA-mediated gene silencing or Hoxc9 overexpression. The produced viral particles, with a multiplicity of infection (MOI) set at 20, were purified and then applied to cancer cells. Subsequent to viral transduction, puromycin was employed as a selective agent to establish cell lines stably expressing either silenced or overexpressed Hoxc9.

### Real time quantitative polymerase chain reaction (RT-qPCR)

Total RNA was isolated from cells 48 hours post transfection utilizing TRIzol reagent (Invitrogen, USA). The sample loading scheme was determined based on the count of gene samples and cells examined. To conform this scheme, a mixture was prepared by combining iTaqUniversal SYBR RT-qPCR mix reagent (Bio-Rad, USA) and primers. All steps in the experimentation adhered to the manufacturers’ guidelines. RT-qPCR was employed to identify the relative expression of Hoxc9 within CMT167 and LLC cells. Hoxc9-specific primers were used for PCR amplification, designed as per the subsequent sequences:

Forward: 5’-CCGACCTGGACCCTAGCAAC-3’.

Reverse: 5’-CCGACGGTCCCTGGTTAAAT-3’.

Actb-specific primers were used for PCR amplification, designed as per the subsequent sequences:

Forward: 5’-GTGACGTTGACATCCGTAAAGA-3’

Reverse: 5’-GCCGGACTCATCGTACTCC-3’

An RT-qPCR apparatus (Roche, USA) was employed to initiate the reaction with a programmed setup. To each well, 1μL of cDNA template was subsequently introduced. Following this, the experiments were conducted in triplicate. The qPCR data was obtained by exporting the results, and the data’s reliability was assessed and examined based on the melting curve analysis.

### Western-blot

To determine Hoxc9 protein expression, Western blotting technique was employed. Initially, RIPA lysis buffer was used to extract total proteins from CMT167 and LLC cells. Protein quantification was carried out using the BCA Protein Assay Kit (Biovision, USA). For protein electrophoresis, a Western-Blotting electrophoresis system (Bio-Rad, USA) was utilized, with 20 micrograms of protein loaded per well. Subsequently, transfer onto a PVDF membrane (Bio-Rad) was executed. Following a blocking phase with 10% skim milk, the membranes underwent antigen-antibody interactions, employing specific antibodies including anti-DDK-tag monoclonal antibody (1:1000, sourced from Abcam), anti-Hoxc9 antibody (1:1000, obtained from Absin), and β-actin (diluted 1:2000; provided by CST). This binding sequence was conducted under persistent shaking, maintained throughout the night at 4° C. Subsequently, on the following day, the membranes were treated with appropriate horseradish peroxidase-conjugated secondary antibodies, specific to each species (diluted 1:5000), and incubated for two hours at ambient temperature. The final visualization of the bands was accomplished using a chemiluminescence detection system.

### Cell viability assay

Cell viability assessment was conducted using the Cell Counting Kit-8 (Takara, China). A cell suspension containing CMT167 and LLC cells in robust growth conditions was prepared, achieving a concentration of 2×10^3^ cells per 100 μl. Subsequently, 100 μL of the cell suspension was dispensed into individual wells of a 96-well plate and positioned within an incubator set at 37° C for initial incubation.

Following this, the subsequent day involved the introduction of 10 μL of CCK-8 solution into each well of a 96-well plate, which was followed by the removal of the preceding culture medium. After undergoing incubation for a duration of 2 hours, the absorbance (OD) was gauged at a wavelength of 450nm using the Multiscan FC microplate reader. The entire procedure was replicated thrice to ensure reliability and consistency.

### Colony formation assay

The evaluation of lung cancer cell proliferation resulting from Hoxc9 knockdown or overexpression was performed using the colony formation assay. Post transfection, CMT167 and LLC cells were digested and then planted into 6-well plates with a cell density of 500 cells per well, in accordance with the characteristics of lung cancer cells. Subsequently, they were placed in a 37° C incubator with 5% CO_2_ and subjected to culturing. When visible clones emerged in most cells within the six-well plate after 10 days of culturing, the culturing process was halted. Following this, formaldehyde was employed for fixation lasting 15 minutes, succeeded by staining with crystal violet for a duration of 10 minutes. Utilizing camera software, images of the clone wells were captured, and clone counting was executed through ImageJ software. Each group underwent a triplicate evaluation of the wells.

### Wound healing assay

Seeded into a 6-well plate were CMT167 and LLC cells. Upon reaching 80% confluence of the cells, the monolayer cells were gently scratched using a sterile micropipette tip. The floating cells were subsequently cleansed with PBS. At 0 hours and 24 hours, the process of wound healing within the scratch was observed. Each condition was evaluated in sets of three wells.

### Cell invasion assay

To conduct the cell invasion assay, 24-well plates with chambers featuring an 8-μm pore size (Corning, Inc.) were utilized. Matrigel was diluted using serum-free medium at a ratio of 1:8. The resultant matrix gel was uniformly applied onto the membrane situated at the base of the Transwell chamber. This assembly was then placed within a 37° C incubator for an incubation period of 2 hours. Following a 48-hour transfection, the lung cancer cells suspension was formulated using serum-free culture medium, with a subsequent adjustment of cell density to 5×10^5^/ml. Simultaneously, 5×10^4^ cells were seeded into each well of the Transwell chamber. In the lower compartment of the 24-well plate, 500-650μL of medium containing 10% FBS was typically introduced. Ultimately, the Transwell chamber was removed, fixed with 4% paraformaldehyde, and subsequently stained using 0.5% crystal violet. Cells present on the upper surface of the filter membrane were gently eliminated using a cotton swab. Employing an optical microscopy, images of cells traversing the invasion chamber were captured.

### Tumor-bearing murine models and treatments

To establish tumor-bearing murine models, 1.0 × 10^6^ LLC cells (infected with lentiviral particle) in 200uL PBS were implanted subcutaneously in the right hind flank of syngeneic BALB/c mice at day 0. Tumor growth was examined at 2, 5, 8, 11, 14, 17 and 20 days. On day 6, 36 mice were randomly assigned to each group, control with IgG isotype (N=6), control with anti-PD-1 (N=6), over expression of Hoxc9 with IgG (N=6), over expression of Hoxc9 with anti-PD-1 (N=6), knockdown of Hoxc9 with IgG (N=6) and knockdown of Hoxc9 with anti-PD-1(N=6). For the groups with anti-PD-1 therapy, an anti-PD-1 mAb (#BE0146, clone: RMP1-14) was administered i.p. every three days, starting at day 5. The control group was injected with IgG control (#BE0089, clone: 2A3) on each injection day. Tumor size was measured by digital calipers every three days and tumor volume was calculated as length×width^2^/2 (mm^3^). Mice were sacrificed and tissues were analyzed at or before the ethical tumor volume limit of 1000 mm^3^.

### Flow cytometry for cell cycle, apoptosis and infiltrated immune cells analyses

To assess the impact of Hoxc9 knockdown on the cell cycle of CMT167 and LLC, flow cytometry analysis was conducted. CMT167 and LLC cells were trypsinized and gathered through centrifugation at 1000rpm for 5 minutes following 48 hours of siHoxc9 or siCtrl transfection. Subsequently, the fixed cells were suspended in pre-chilled 75% ethanol and placed in a refrigerator at 4° C overnight. Adhering to the guidelines from the flow cell cycle kit (Biyuntian, Shanghai), the cells were thoroughly mixed, then filtered through the membrane and transferred to a 5ml flow tube.

1X Binding Buffer was prepared according to the instructions for flow cytometry. Collect transfected lung cancer cells and resuspend the cells with 1X staining buffer to a cell concentration of 5 x 10^6^ cells/mL. Add 5μL fluorescent dye-conjugated Annexin V and incubate for 10-15 min at room temperature. The cells were then resuspended in staining buffer. 5μL propidium iodide (PI) dye was added and incubated on ice for 15 minutes. Flow cytometry was used to read the data.

Mice were sacrificed by cervical dislocation, and tumors were harvested to prepare single cell suspensions. The single-cell suspensions of tumor tissue were generated with tumor dissociation kit, mouse (Miltenyi Biotec, Germany) according to manufacturer’s instructions. Single cell suspensions were stained with fixable viability dye (Fixable Viability Stain 780, BD Pharmingen, USA) at room temperature for 10 minutes to rule out dead cells. Cell surface markers were assessed using the following antibodies incubated with cell suspensions in the dark at 4° C for 30 minutes: CD45 (BD Pharmingen), CD3 (1:20; #100205, #100218, Biolegend), CD4 BV605 (BD Pharmingen), IFN-γ (1:20; #505808, Biolegend). The flow cytometry data were acquired on Beckman Cytoflex (Beckman Coulter, USA) and the results were analyzed with FlowJo V.10.6.1 software (TreeStar).

### Study population, treatment and assessments

Prospective enrollment took place at Shanghai Pulmonary Hospital, involving patients with advanced LUAD. Inclusion criteria encompassed age between 18 and 70, confirmed histologically or cytologically as stage IV LUAD (as per the 8th edition of the International Association for the Study of Lung Cancer Staging Handbook in Thoracic Oncology), lack of EGFR and ALK alterations, ECOG PS score of 0 or 1, no prior systemic therapy, presence of at least one measurable lesion following RECIST v1.1, and a projected life expectancy of over 3 months. Complete baseline characteristics or radiologic images, including chest CT, brain MRI, and PET-CT scan, were evaluated by two specialized radiologists. Before commencing the first-line combination of PD-1 blockade and chemotherapy, tumor samples were acquired through image-guided percutaneous lung biopsy. PD-L1 expression was evaluated as part of the initial screening procedure at the central pathology laboratory within our institution. This evaluation hinged on the application of the Tumor Proportion Score (TPS), a metric that gauges the proportion of viable tumor cells displaying partial or complete membrane staining, regardless of the intensity of staining. The resulting values from this assessment were routinely classified into three distinct groups: TPS <1%, TPS 1-49%, and TPS ≥50%. The study was approved by the ethics committee of Shanghai Pulmonary Hospital and was conducted in accordance with the Declaration of Helsinki (as revised in 2013). All participants signed informed consent forms before the initiation of this research undertaking.

Treatment was administered until radiographic progression, the occurrence of intolerable toxic effects, a determination made by the investigator, or the voluntary withdrawal of consent by the patient. If toxicity could be definitively attributed to a single agent, that specific drug could be discontinued. Tumor imaging sessions were planned at weeks 6 and 12, followed by intervals of 9 weeks until week 48, and subsequently, at 12-week intervals. The assessment of response was conducted in accordance with RECIST, version 1.1 [25]. Additionally, there was a regular patient contact every 12 weeks to evaluate survival outcomes during the follow-up period. The primary endpoints encompassed OS and PFS. OS indicated the time from randomization to death resulting from any cause. PFS denoted the time from randomization to either disease progression or death, whichever transpired first, as evaluated by a blinded, independent central radiologic review. Secondary endpoints included the response, the duration of response, and safety.

### Immunohistochemical staining

Paraffin-embedded lung cancer tissue samples were sliced to 4um thickness and affixed onto slides for subsequent deparaffinized hydration. The tissue was then restored to its postfixed state, exposing the antigen for precise antibody binding. Following antigen restoration under high temperature and pressure, endogenous peroxidase was neutralized through a 10-minute treatment with hydrogen peroxide. Blocking utilized goat-derived animal serum. Antibodies were incubated with sections before immunohistochemical (IHC) staining. The slices were covered with diluted HOXC9, CD4, CD8, CD68 and FOXP3 (Abcam, Cambridge, MA, USA) mouse monoclonal antibodies, and this covering occurred in a humidified chamber for 2 hours at 37° C. After an overnight 4° C block, sections were fully covered with a secondary antibody and incubated at 37° C for 30 minutes. Subsequent DAB (Abcam, Cambridge, MA, USA) coloration was meticulously controlled. Slices were sealed with neutral gum, air-dried in a fume hood, and then microscopically analyzed for images. Staining assessments were performed simultaneously by two independent observers, scoring each stained section and reaching a consensus. According to the median percentage of positive cell proportion in each marker, the cut-off values of positive status were calculated.

### Statistical analysis

Statistical analyses were performed using the aforementioned online databases and R software (version 3.6.3). Data are presented as mean ± SD. For the statistical analysis of experimental data, GraphPad Prism 9.5.1 (GraphPad Software; Dotmatics) was employed. Unpaired Student’s t test and Wilcoxon rank-sum test and one-way ANOVA tests (with LSD as post hoc test) were used to calculate the significance of differences in data between and among groups. P<0.05 was considered to indicate a statistically significant result.

### Availability of data and materials

Bioinformatics datasets presented in this study can be found in online repositories, and the datasets used and/or analyzed during experiments are available from the corresponding author on reasonable request.

### Consent for publication

All authors have approved the manuscript for submission.

## RESULTS

### HOXC9 is overexpressed and correlated with prognosis in LUAD

**Figure 1:**
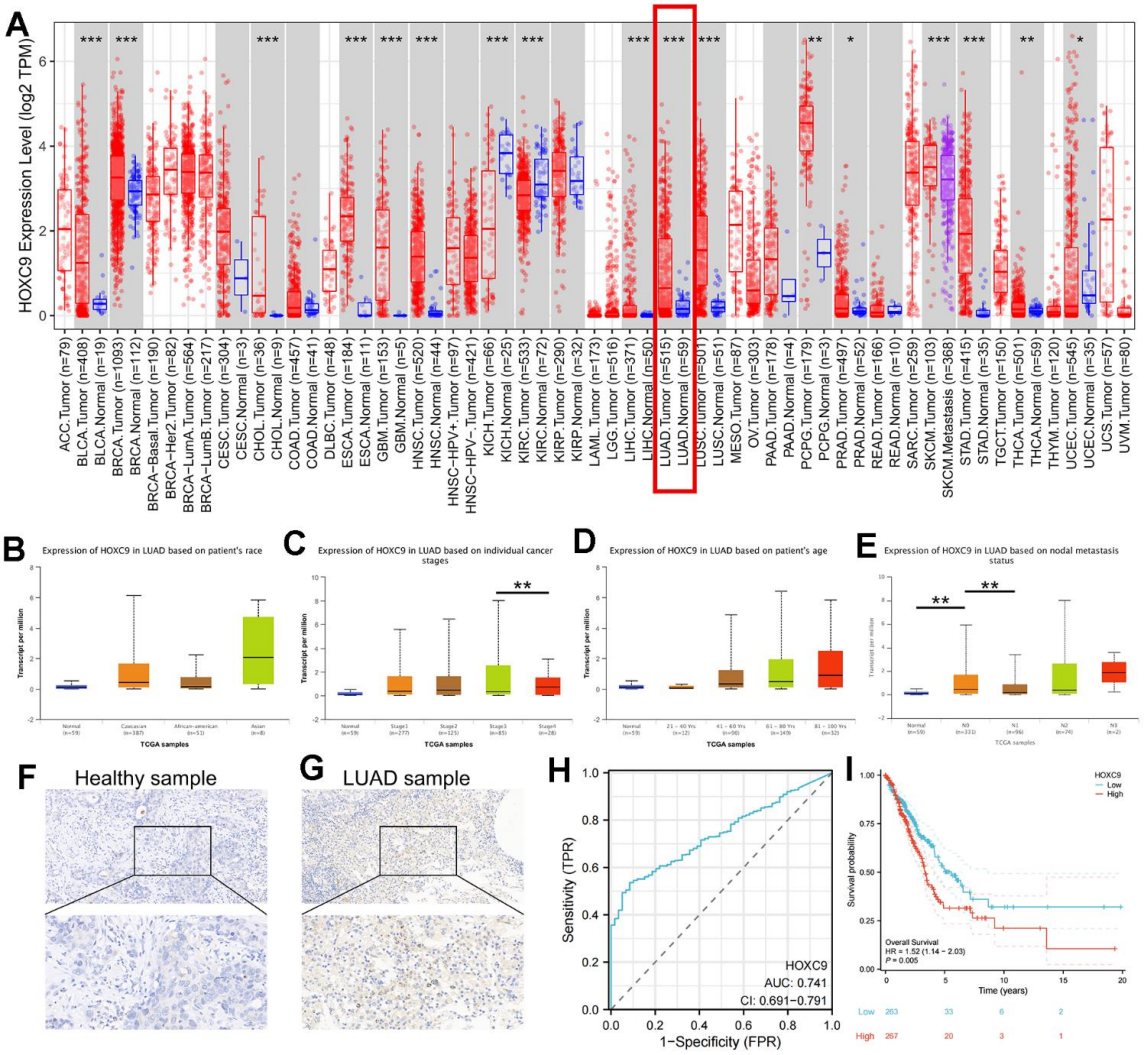
**Analyses of HOXC9 expression and the diagnostic and prognostic value in LUAD.** (**A**) The expression distribution of HOXC9 in tumor tissues and normal tissues with TIMER2, respectively; (**B**–**E**) The correlation between HOXC9 expression and clinical variables of LUAD; (**F**) The HOXC9 protein level in adjacent healthy tissue; (**G**) and in the tumor tissue; (**H**) The diagnostic value of HOXC9 in LUAD; (**I**) The OS analysis of HOXC9 in LUAD patients. (*, P<0.05; **, P<0.01; ***, P<0.001).

Firstly, we assessed HOXC9 expression in pan-cancer data from TCGA and GTEx. The results showed that HOXC9 was significantly higher in 14 types of tumors than corresponding normal tissues including BLCA, BRCA, CHOL, ESCA, GBM, HNSC, LUAD, LUSC, PCPG, PRAD, SKCM, STAD, THCA, and UCEC, with TIMER2 pan-cancer analysis (all P<0.05) (Figure 1A). Then, the correlations between HOXC9 expression level and different clinical variables in LUAD were analyzed via UALCAN tools, which indicated higher HOXC9 expression was significantly associated with higher individual stages and tumor grade (Figure 1B–1E). To confirm this finding, we compared HOXC9 protein level between adjacent normal and cancerous tissues from clinical LUAD samples, and also found it was upregulated in tumor sample (Figure 1F, 1G). Moreover, the receiver operating curve, with the aera under the curve of 0.741 (95%CI 0.691-0.791), indicated a high diagnostic value of HOXC9 in LUAD, as is shown in Figure 1H. A survival analysis was performed, showing high expression of HOXC9 was associated with shorter OS (HR=1.52, 95%CI 1.140-2.030, P=0.001) in LUAD (Figure 1I).

At the facet of genomic exploration, through the online database cBioPortal, HOXC9 genetic mutations and alterations landscape were investigated in various tumor samples from TCGA datasets (Supplementary Figure 1A). The main type of genetic alterations in HOXC9 was “mutation”, which were observed in bulk of TCGA cancers, and the “amplification” was the second most common. The frequency of HOXC9 alteration in LUAD patients was mRNA high. With COSMIC online tool, the overview of the types of mutation was observed. The primary mutation type was missense substitution (52.43%), and the primary substitution mutation type was C>T (33.22%) (Supplementary Figure 1B).

### HOXC9 expression is related to repressive immune cells and play a hazard role in responses to immunotherapy

**Figure 2:**
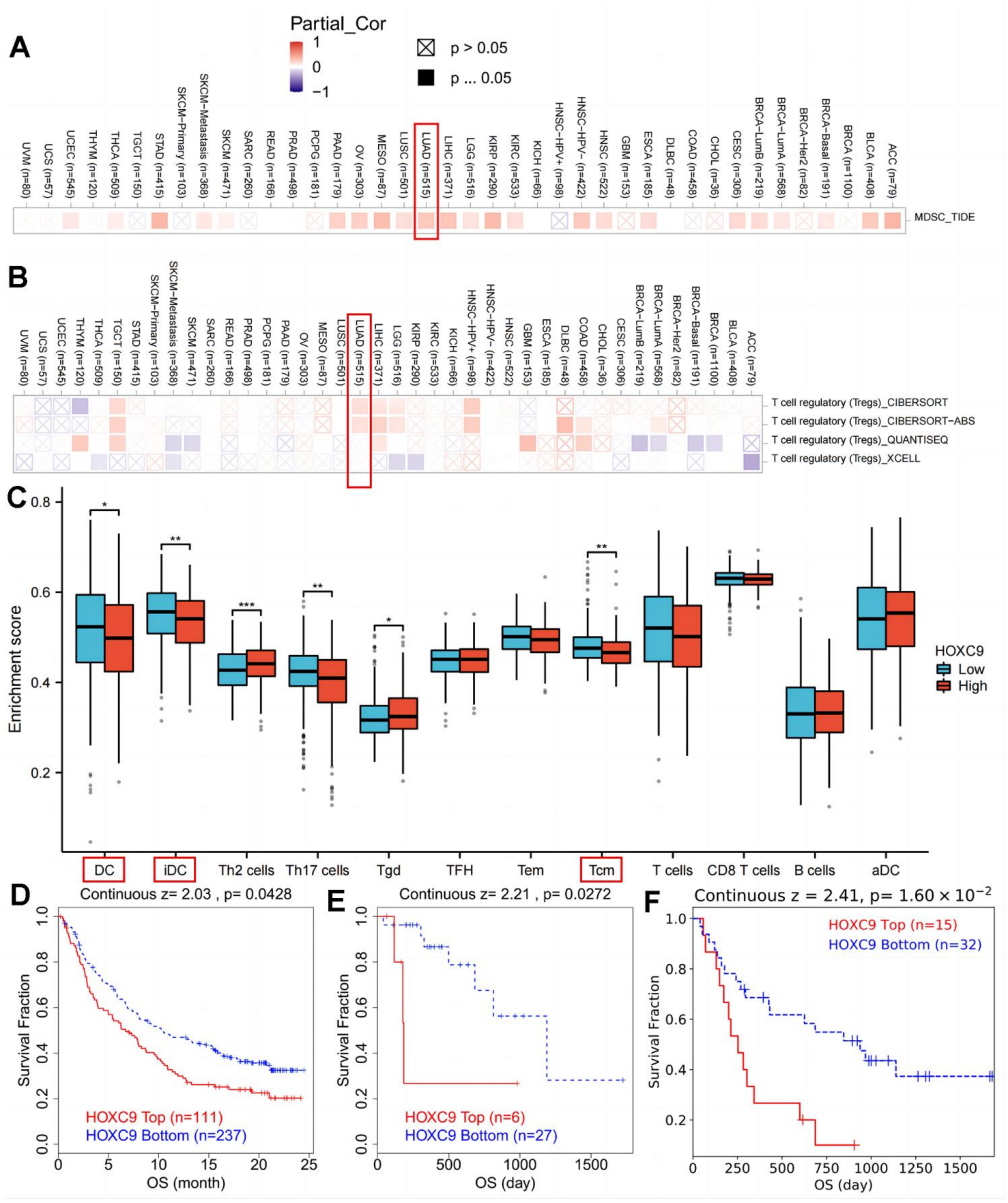
**The correlation analysis between HOXC9 level and suppressive immune cells among cancers.** (**A**) HOXC9 was significantly related to MDSC in LUAD. (**B**) HOXC9 was significantly related to Tregs in LUAD. (**C**) patients with high HOXC9 possessing lower DC and Tcm infiltrations. (**D**) HOXC9 expression was related to shorter OS of bladder cancer. (**E**) kidney cancer. and (**F**) melanoma treated with ICIs. (*P<0.05, **P<0.01, ***P<0.001).

The relationship between HOXC9 expression and immune repressive cells’ infiltration (myeloid-derived suppressor cells and Tregs) was explored in Figure 2A, 2B. To further explore the role of HOXC9 in TME, we conducted a correlation analysis of HOXC9 expression with other immune infiltration cells in LUAD, which demonstrated that patients with relatively high HOXC9 possessed less dendritic cells and T central memory cells (Figure 2C). To further verify the negative role of HOXC9 in responses to immunotherapy, the data from TIDE showed relatively higher HOXC9 was related to worse OS in bladder cancer (P=0.043, Figure 2D), kidney cancer (P=0.027, Figure 2E), and melanoma (P=0.016, Figure 2F) treated with ICIs.

### Knockdown of Hoxc9 reduces the proliferative capacity of in murine NSCLC cells

**Figure 3:**
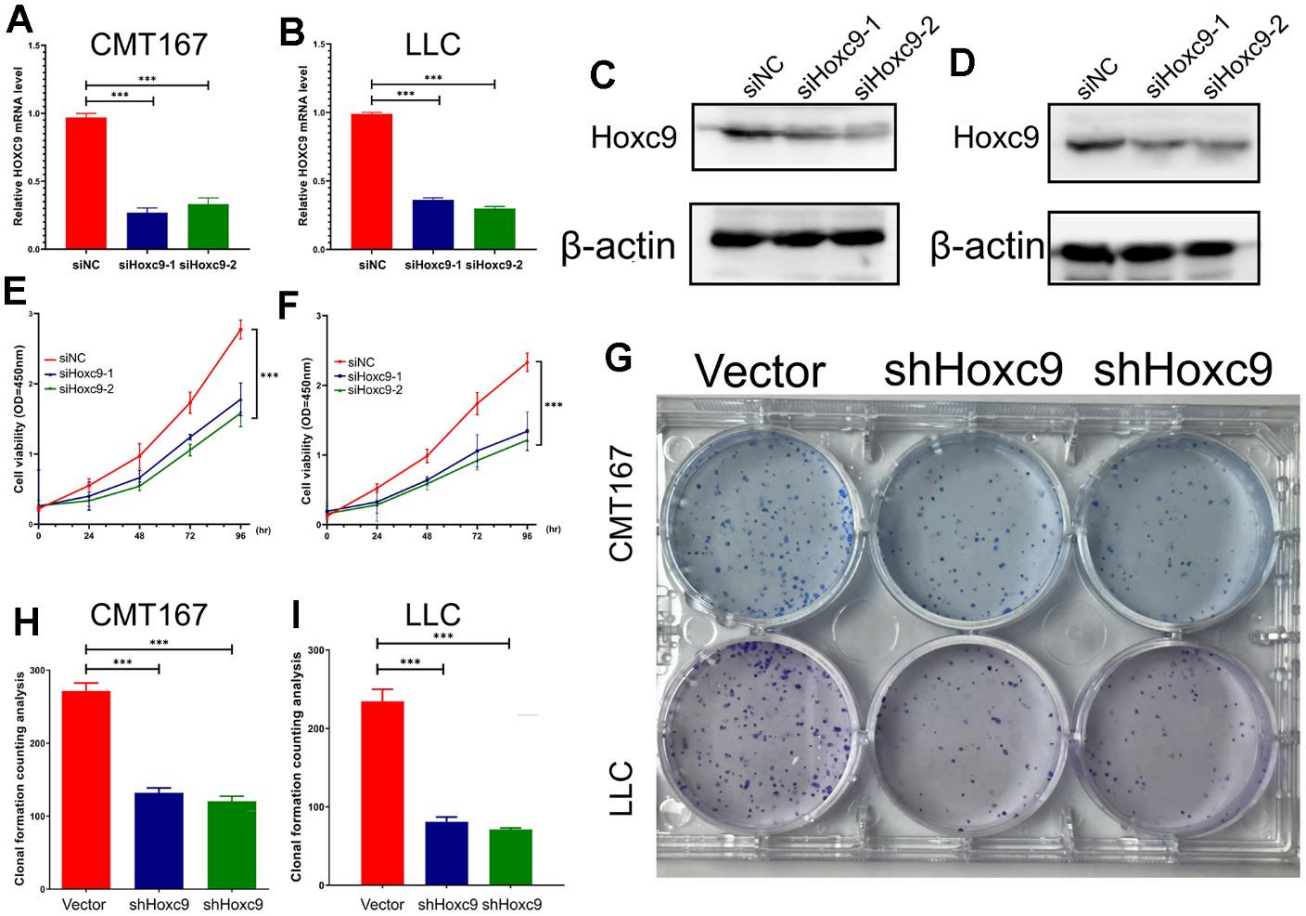
**Confirmation of Hoxc9 knockdown and its impacts on viability and proliferation of CMT167 and LCC cells.** (**A**, **B**) The knockdown level of Hoxc9 was detected by RT-qPCR. (**C**, **D**) The knockdown level of Hoxc9 was detected by western blot. (**E**, **F**) The cell viability was significantly impaired under Hoxc9 knockdown. (**G**–**I**) The influenced proliferation competence was detected with colony formation after 10 days. (*P<0.05, **P<0.01, ***P<0.001).

Taken together, HOXC9 is an oncogene that drives tumorigenesis and progression in lung cancer and can lead to poor prognosis by activating cell proliferation and causing immune dysfunction. We then performed a series of *in vitro* experiments to verify that knockdown of Hoxc9 at the cellular level could inhibit its malignant biological behavior. Subsequently, CMT167 and LLC cell lines were transfected with siRNA against Hoxc9. The knockdown efficiency of siHoxc9-1 and siHoxc9-2 was assessed by RT-qPCR, and the results showed that siRNA effectively reduced the mRNA level of Hoxc9 in CMT167 and LLC cells (Figure 3A, 3B). Subsequent western blot experiments also verified that the protein expression level of Hoxc9 was significantly reduced after transfection of siRNA (Figure 3C, 3D). CCK8 cell proliferation assay was used to detect the effect of knockdown of Hoxc9 on cell proliferation. Compared with siNC (si negative control) group, the proliferation rate of CMT167 and LLC cells in Hoxc9 knockdown group was significantly reduced (Figure 3E, 3F, P<0.001). Colony formation assay was used to detect the effect of Hoxc9 knockdown on cell colony formation. Compared with vector group, the number of CMT167 and LLC colony groups in Hoxc9 knockdown group was significantly reduced (Figure 3G, 3I, P<0.001). The results showed that knockdown of Hoxc9 expression reduced cell proliferation and significantly inhibited the colony formation ability of CMT167 and LLC cells.

### Knockdown of Hoxc9 promoted apoptosis and cell cycle arrest

**Figure 4:**
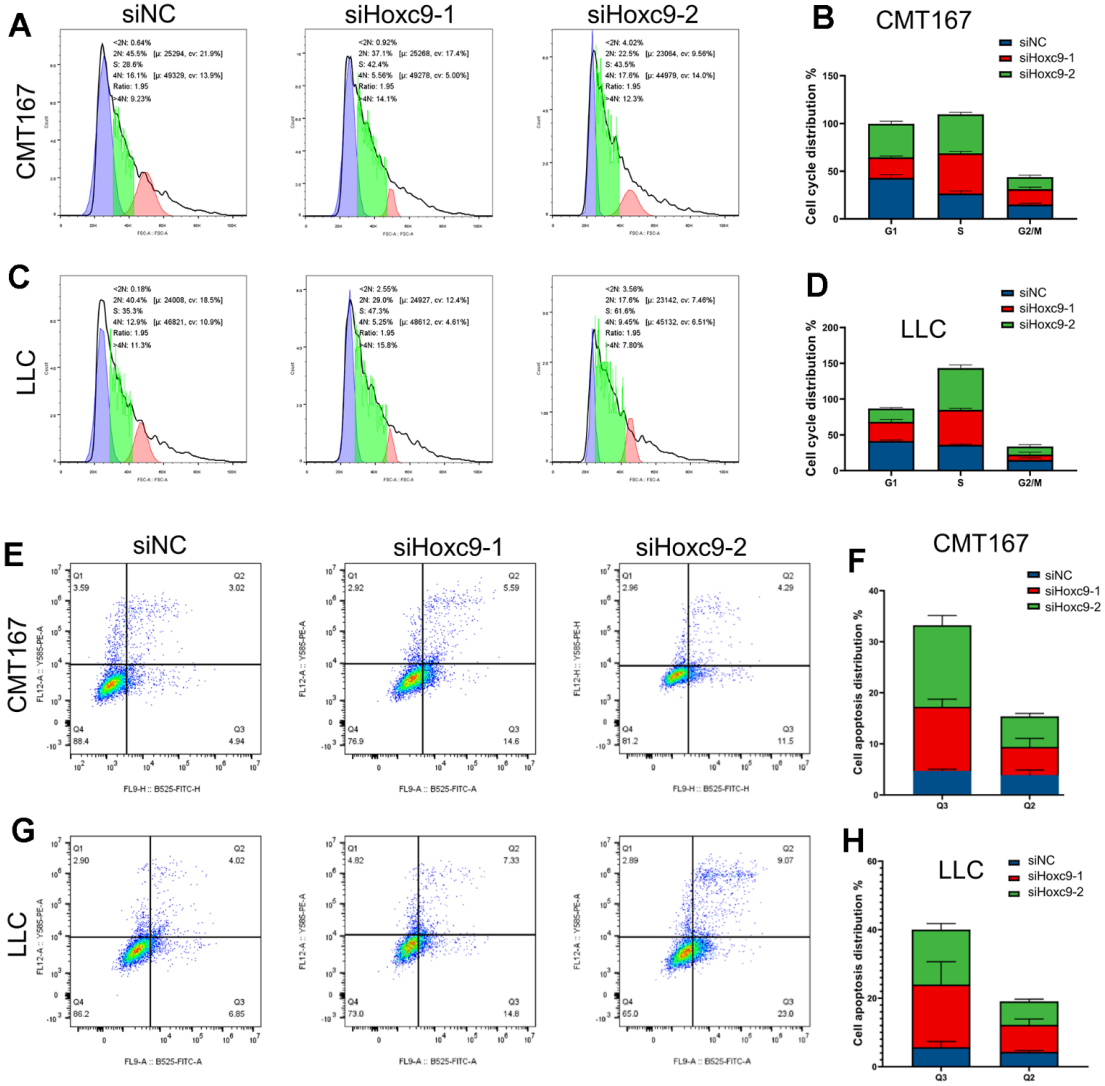
**Hoxc9 knockdown arrested cell cycle and promoted cell apoptosis.** (**A**–**D**) Hoxc9 knockdown arrested cell cycle of CMT167 cells and LCC cells. (**E**–**H**) Hoxc9 knockdown promoted cell apoptosis of CMT167 cells and LCC cells. (*P<0.05, **P<0.01, ***P<0.001).

Flow cytometry was used to analyze the effect of Hoxc9 knockdown on cell cycle and apoptosis. The results of cell cycle experiments suggested that the number of cells in S phase were significantly increased and the number of cells in G1 phase was decreased in the two Hoxc9 knockdown groups compared with the siNC group (Figure 4A–4D, P<0.001), indicating that the proliferation ability was significantly impaired after blocking Hoxc9 in CMT167 and LLC cells. Subsequently, flow cytometry PI/Annexin V was used to analyze the effect of Hoxc9 knockdown on the apoptosis of CMT167 and LLC lung cancer cells. The results suggested that the number of early apoptotic cells was significantly increased in the two Hoxc9 knockdown groups compared with the siNC group (Figure 4E–4H, P<0.001). The results showed that knockdown of Hoxc9 expression caused lung cancer cell apoptosis, and this biological effect may be related to cell cycle S phase arrest.

### Knockdown of Hoxc9 reduces the migrative and invasive capacity of in murine NSCLC cells

**Figure 5:**
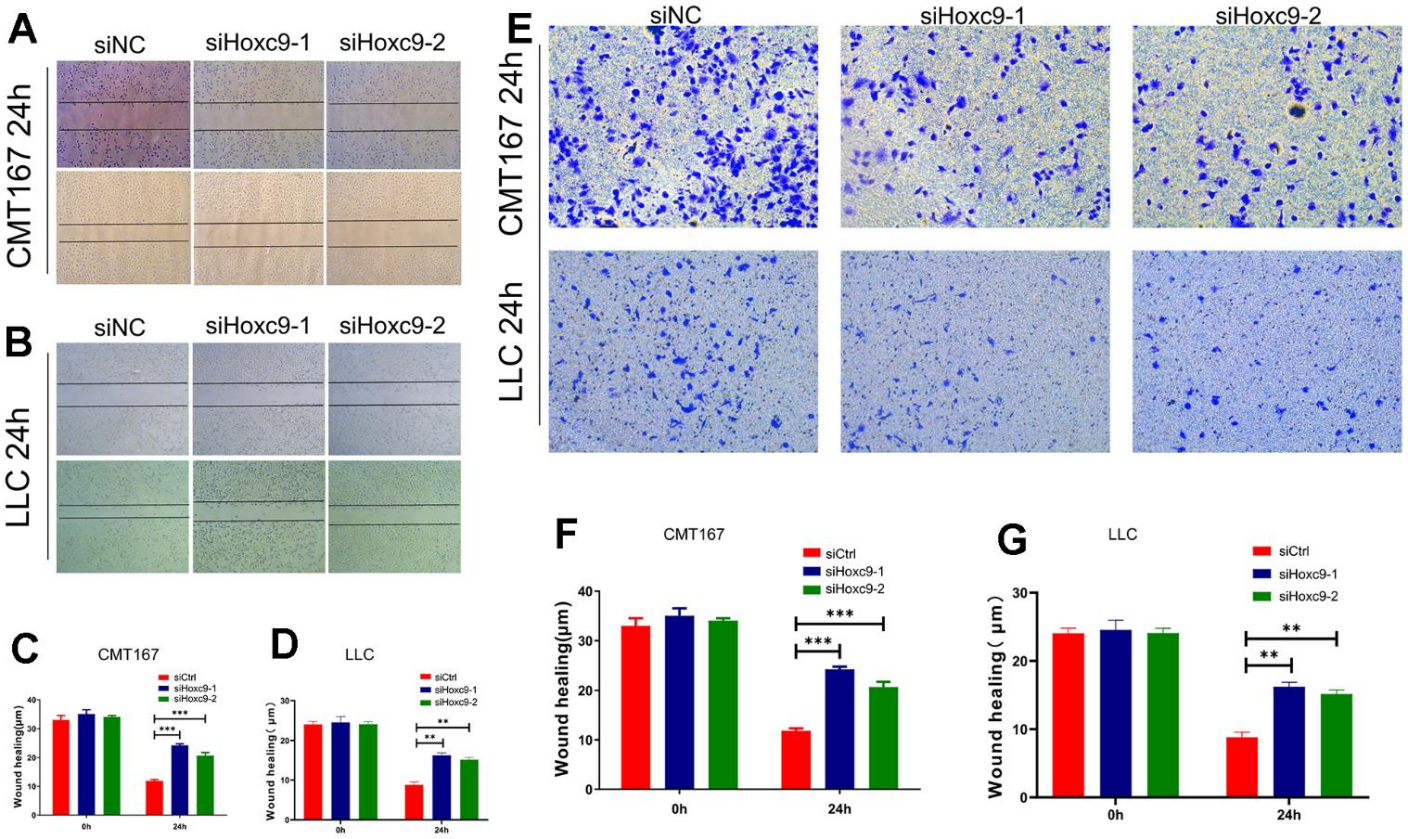
**Hoxc9 knockdown inhibited the migration and invasion of tumor cells.** (**A**) Remaining length of wound of CMT167 under knockdown of Hoxc9 was significantly longer compared to control (200 μm). (**B**) Remaining length of wound of LLC under knockdown of Hoxc9 was significantly longer compared to control (200 μm). (**C**, **D**) The statistical analysis of above assays. (**E**–**G**) Count of migrated CMT167 and LCC cells was significantly decreased after knockdown of Hoxc9. (*P<0.05, **P<0.01, ***P<0.001) (100 μm).

As for the impacts of Hoxc9 knockdown on migration and invasion of CMT167 and LLC, we conducted the wound healing and Transwell assays to investigate. After 24h, cells transfected with siHoxc9 showed significantly wider remaining wound than siNC groups (Figure 5A–5D), and showed the evidently less penetrated cells than siNC groups (Figure 5E–5G).

### Overexpression of Hoxc9 promoted cell proliferation and migration in lung cancer cell lines

**Figure 6:**
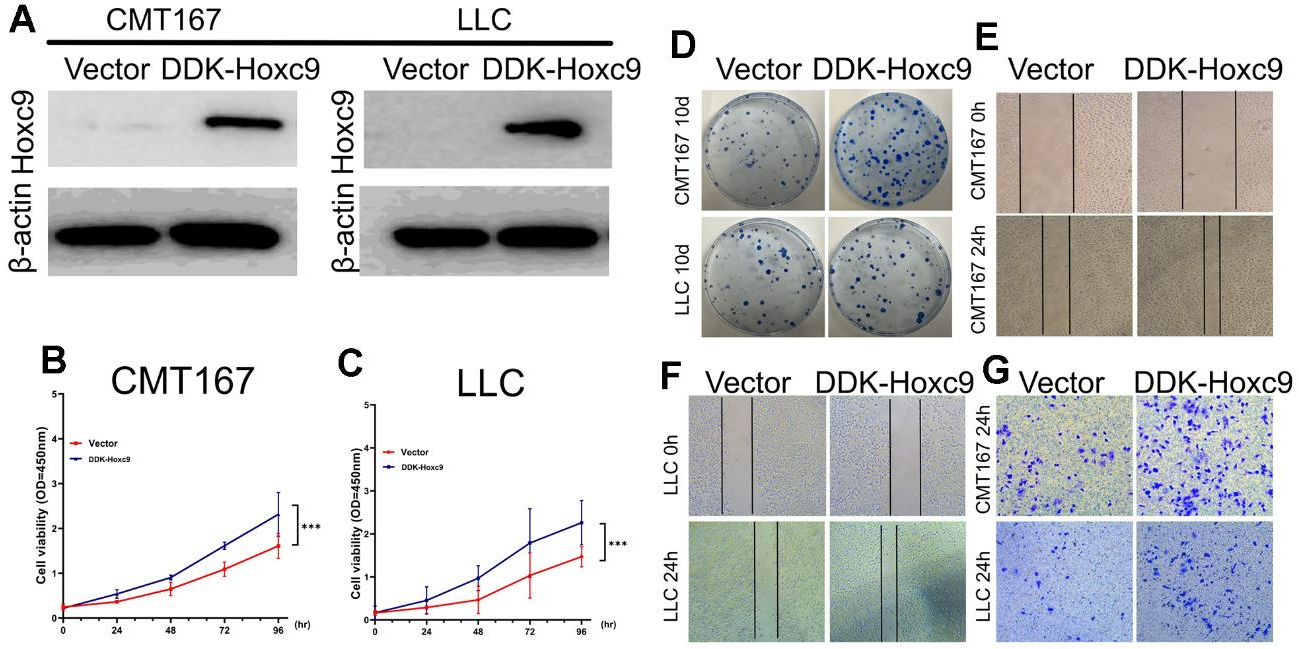
**Overexpression of Hoxc9 elicited a promotive role in tumor cells progression.** (**A**) Confirmation of Hoxc9 overexpression in two cell lines with WB. (**B**, **C**) Significantly higher cell viabilities were observed in the DDK-Hoxc9 groups of CMT and LCC. (**D**–**F**) Overexpression of Hoxc9 resulted in a more counts of colony and wider remaining wound (200 μm). (**G**) Overexpression of Hoxc9 resulted in more invasive counts of cells (100 μm). (*P<0.05, **P<0.01, ***P<0.001).

The functional of Hoxc9 overexpression in lung cancer was determined by *in vitro* cell experiments. Ddk-tagged Hoxc9 overexpression vector was constructed and transfected into CMT167 and LLC cells. Western blot analysis confirmed that Hoxc9 was overexpressed in CMT167 and LLC cells after plasmid transfection (Figure 6A). Overexpression of Hoxc9 promoted lung cancer cell proliferation (Figure 6B, 6C) and colony formation (Figure 6D). In CMT167 and LLC cells, Hoxc9 overexpression promoted increased migration of lung cancer cells (Figure 6E, 6F) and invasion of lung cancer cells (Figure 6G). Overall, overexpression of Hoxc9 promotes lung cancer proliferation, colony formation, and tumor cell invasion and progression.

### Differential Hoxc9 expression levels mattered tumor growth and CD8+ T cells infiltration with or without PD-1 blockade

**Figure 7:**
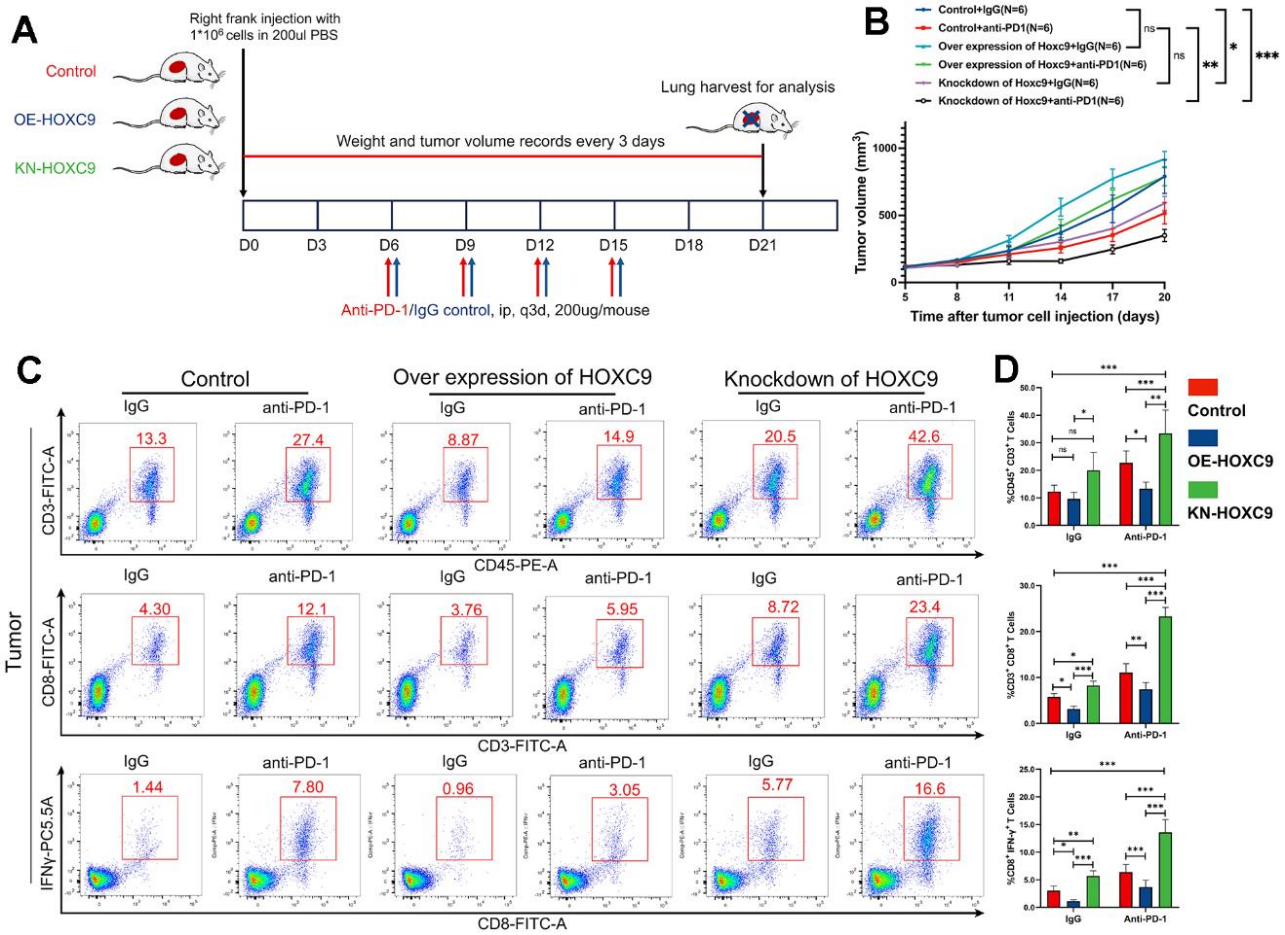
**Combination of Hoxc9 inhibition and PD-1 blockade enhanced the tumor growth and improved CD8+ T cell infiltration.** (**A**) Diagram of *in vivo* experiment design. (**B**) Tumor growth curves of different groups. (**C**, **D**) More effective CD8+ T cells were observed in Hoxc9 knockdown groups compared to control groups under IgG/PD-1 blockade. (*P<0.05, **P<0.01, ***P<0.001).

With the establishment of tumor-bearing murine models, we verified the hypothesis that Hoxc9 expression level impacted tumor growth and sensitivity to PD-1 blockade, and the diagram was shown at Figure 7A. As shown in Figure 7B, a notable reduction in tumor growth rate was evident within the Hoxc9 knockdown group compared to the control group, both under IgG isotype conditions (P<0.05) and following PD-1 blockade intervention (P<0.01) (the tumor models photograph was in Supplementary Figure 2). Overall, while the efficacy of PD-1 blockade exhibited a positive trend over Hoxc9 knockdown, statistical significance was not achieved. Intriguingly, the overexpression of Hoxc9 nearly counteracted the effects of PD-1 blockade, as observed in the control group with IgG isotype. As anticipated, the most substantial disparity in tumor sizes was observed between the control group treated with IgG and the Hoxc9 knockdown group subjected to PD-1 blockade (P<0.001).

The present study involved the disaggregation of mouse tumor tissues into individual cells for subsequent flow cytometry analysis. The outcomes of the sorting process are illustrated in Figure 7C. In the isotype control group, there was no statistically significant augmentation observed in tumor-infiltrating CD3+ T cells. However, upon the attenuation of Hoxc9 expression, a noteworthy increase was noted in activated CD8+ T cells (P<0.05) and IFN-γ production (P<0.01) (Figure 7D). Conversely, the overexpression of Hoxc9 elicited an opposing effect. Additionally, with the treatment of PD-1 blockade, concurrent with tumor growth dynamics, the downregulation of Hoxc9 exhibited a pronounced augmentation in CD3+ T cell infiltration, activation of CD8+ T cells, and an upsurge in IFN-γ production (P<0.001). These findings collectively suggest a potential synergistic antitumor immune response through the concomitant modulation of immune checkpoints and Hoxc9.

### HOXC9 expression improves the predictive power for response to PD-1 blockade plus chemotherapy and survival in LUAD

**Figure 8:**
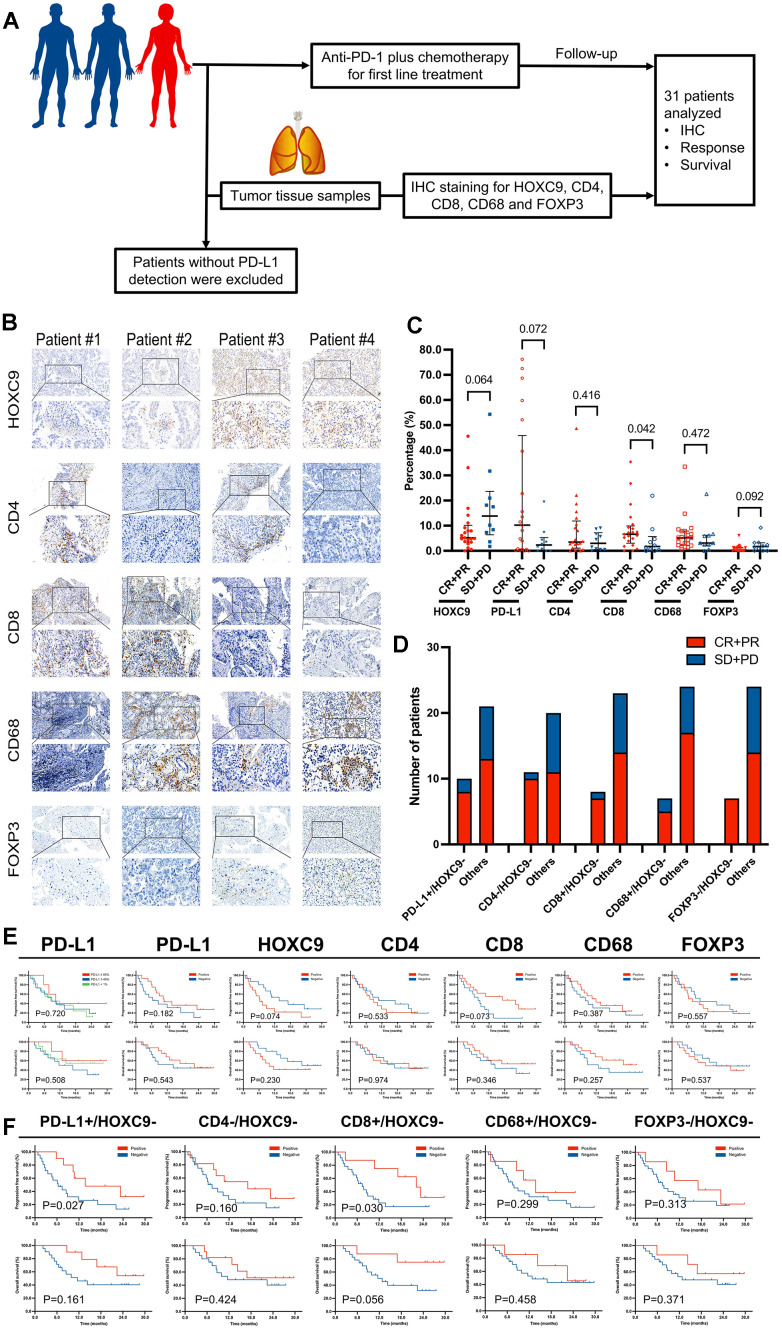
**The predictive value of HOXC9 and TME markers for LUAD patients treated with PD-1 blockade plus chemotherapy.** (**A**) Diagram of clinical samples selection and analysis. (**B**) IHC analysis of 5 proteins based on samples from 4 patients with different level of HOXC9. (**C**) The different distributions of responder population under 5 types of TME marker. (**D**) Analysis of predictive value of combination of HOXC9- and other markers. (**E**) PFS and OS analysis based on different status of markers. (**F**) PFS and OS analysis based on different combinations. (*P<0.05, **P<0.01, ***P<0.001).

Three officially approved predictive biomarkers, including PD-L1 expression, TMB, and MSI, guide the clinical application of anti-PD-1/PD-L1 monotherapy with moderate predictive performance [3, 8]. However, effective predictive markers for ICIs combined with chemotherapy remain underreported [8, 9]. Within the TME, characterized by distinct subpopulations of immune cells such as CD3+, CD8+, CD68+, and FoxP3+ cells, as well as other known or potential molecules, exists a diverse array of predictors that warrants continuous exploration [3, 12]. To investigate the predictive value of HOXC9 expression in the context of PD-1 blockade plus chemotherapy, we prospectively enrolled advanced LUAD patients with no prior systemic treatment for first-line immunochemotherapy, and baseline biopsy samples were obtained for IHC analysis. Finally, from January 2021 to March 2021, 31 patients treated with anti-PD-1 plus chemotherapy from our center were included in the final analysis (Figure 8A). The baseline characteristics of the participants were presented in Supplementary Table 1. The positive and negative expression of HOXC9, CD4, CD8, CD68, and FOXP3 divided by the median were presented in Figure 8B. Our initial assessment involved examining correlations between individual TME marker expressions and objective responses. Notably, responders exhibited significantly higher CD8 positive expression, as indicated in Figure 8C. PD-L1 expression demonstrated an increasing trend among responders, the expressions of HOXC9 and FOXP3 displayed a decreasing trend, but statistically insignificant. Worth mentioning is the substantially low overall expression of FOXP3. The clinical practice widely employs a trichotomy of PD-L1 TPS using cutoff values of 1% and 50%. Unfortunately, this trichotomy’s predictive capacity for treatment response and survival proved to be even inferior to the dichotomy of PD-L1 TPS based on median values.

We then conducted a focused investigation to examine the correlations between various combinations of HOXC9 and other markers and their impact on objective response. The results revealed a distinct differentiation between responders and non-responders, as illustrated in Figure 8D. Subsequently, we carried out a more detailed exploration into the prognostic value of individual markers and combinations of HOXC9 with other markers. Notably, none of the individual markers exhibited predictive ability for survival, as depicted in Figure 8E. However, patients characterized as PD-L1+/HOXC9- and CD8+/HOXC9- both showed a notably extended PFS (P=0.027 and P=0.030), as indicated in Figure 8F. In general, the inclusion of HOXC9 tended to enhance the differentiation of patients with differential prognosis. Consequently, the expression of HOXC9 generally enhances the predictive accuracy for both treatment response and survival outcomes when PD-1 blockade is combined with chemotherapy in cases of LUAD.

## DISCUSSION

The utilization of PD-1/PD-L1 inhibitors, combined or not combined with chemotherapy, has become the standard of care as first-line treatment for patients with locally advanced or metastatic NSCLC. Nonetheless, the identification of dependable biomarkers remains a notable deficiency, particularly in the context of ICIs complemented by chemotherapy. We proudly present, to the best of our knowledge, the pioneering investigation unveiling the predictive potency of HOXC9 in the realm of immunotherapy for LUAD patients. This groundbreaking study seamlessly amalgamates bioinformatics scrutiny, *in vitro* and *in vivo* experiments, alongside clinical data pertaining to responses to immunotherapy and patient survival, culminating in an exhaustive and insightful analysis.

HOXC9 was previously reported to play a promotive role in oncogenesis [18], repressive TME [19], as well as the chemotherapy resistance of bladder cancer [26] High HOXC9 expression in LUAD indicated better OS and DFS, whereas HOXC9 expression levels were not associated with OS or DFS in lung squamous cell carcinoma (LUSC), which may originate from differential pattern of immune cell infiltration [19]. The irrelevance between HOXC9 and survival in LUSC was verified by us and the no positive correlation was found between HOXC9 expression and Treg in LUSC (Figure 2B). Thus, the present study focused on LUAD. In line with previous studies, we found that HOXC9 was related to oncogenesis, stage and OS in LUAD (Figure 1). *In vitro* experiments further demonstrated that regulation of Hoxc9 expression significantly impacted the proliferation, migration and invasion of two murine tumor cell lines, CMT167 and LLC (Figures 3–6). Moreover, HOXC9 level plays a hazard role in the response to ICIs among multiple cancers, which might be attributed to its negative relationship with antitumor immune function (Figure 2). However, its specific function under immunotherapy in LUAD is rarely investigated.

Our study pioneers the exploration of Hoxc9 expression’s impact within the TME and its influence on survival outcomes in the context of ICIs therapy, employing *in vivo* experiments. As for tumor volume, with or without ICIs treatment, knockdown of Hoxc9 significantly inhibited the growth of tumors. A “hot” TME is crucial for ICIs to work, characterized with the presence of activated TIL landscapes [27, 28], thus T cells sorting was used to reflect the extent of immune activation. Accordingly, T cell sorting was employed as a measure of immune activation. In line with tumor growth patterns, the downregulation of Hoxc9 was associated with a significant increase in activated CD8+ T cells and IFN-γ production. Notably, tumors subjected to both Hoxc9 knockdown and ICI treatment exhibited the most substantial growth inhibition and pronounced activation of CD3+/CD8+/IFN-γ+ T cells. This observation suggests a potential synergistic effect of PD-1 blockade and HOXC9 inhibition in enhancing the therapeutic efficacy in LUAD patients.

Admittedly, targeting HOXC9 or related pathways [18, 19], represents a potential therapeutic strategy for LUAD or NSCLC, the development of targeted drugs is a long way off. In our view, its predictive value for response to ICIs treatment and survival deserves more attention. There is almost a consensus that a single marker is difficult to predict outcomes accurately in immunotherapy, due to patient heterogeneity and the complexity of the antitumor immunity [29], accumulating evidence showed that incorporating distinct biomarkers and even multi-omics is the key to developing robust predictors of response. The TME features have long been considered as favorable predictive markers for immunotherapy, which was firstly classified into four different subtypes according to the PD-L1 expression and TILs status [30]. In terms of advanced NSCLC, several studies have found that differential types of TME, based on PD-L1 expression and CD8+ TILs or CD68+ macrophages, could accurately predict treatment responses and survival in ICI monotherapy [31–33]. A recent prospective study by Grell et al. also revealed combining FOXP3 and CD68 could better predict both PFS and OS than FOXP3 alone in NSCLC patients treated with ICI monotherapy [34]. More strikingly, multiplex immunohistochemistry/immunofluorescence have been developed to combine the TME features, demonstrating robustly predictive value [8, 32, 33].

As for ICIs combined with chemotherapy, only two studies have reported valid results. Expression of major histocompatibility complex class II (MHC-II) antigen presentation pathway expression was recently reported to successfully identify patients most likely to benefit in non-squamous NSCLC [9]. The other study preliminarily revealed the predictive value of CD8/PD-L1 or CD68/PD-L1 co-expression for camrelizumab plus chemotherapy as first-line treatment in patients with locally advanced or metastatic NSCLC [8]. The present study also found existing single marker couldn’t reliably predict the response to immunochemotherapy and outcomes. However, the addition of HOXC9 tended to better distinguish patients with differential responses and prognosis, and the PD-L1+/HOXC9- and CD8+/HOXC9- patients correlated with significantly longer PFS. Thus, in general, results from the small sample suggest HOXC9 expression could improve the predictive power for response to PD-1 blockade plus chemotherapy and survival outcomes in LUAD.

Several limitations should be acknowledged. First, public data on HOXC9 in lung cancer immunotherapy cohort are missing. Second, our research focused on phenotypes, so more rigorous *in vivo* experiments are needed to confirm the current findings and help to reveal detailed characteristics of HOXC9 in LUAD. Third, the TME in the stomal area couldn’t be evaluated as all available samples were biopsy specimens from tumor area. Lastly, although our study was prospectively designed and baseline characteristics were well balanced, considering the limited sample size, the present results should be cautiously interpreted and still need further independent validation with large sample size.

The combination of various biomarkers, including patient characteristics, imaging, pathology, peripheral blood, and genomic data, holds potential in guiding holistic treatment approaches. The integration of machine learning with multimodal features emerges as a promising method for predicting treatment responses [35]. Further research would investigate the specific cell subtypes expressing HOXC9 in LUAD microenvironment with single-cell RNA sequence, identify the downstream interactive molecules, and clarify the detailed regulatory role of HOXC9 in LUAD immunotherapy.

## CONCLUSIONS

In summary, this study integrated data from multiple bioinformatic public databases, *in vitro* and *in vivo* experiments, and baseline biopsy samples from our center to identify a potential biomarker for ICIs plus chemotherapy in LUAD. The integration of HOXC9 expression profiles with other clinically pertinent markers holds significant potential as a comprehensive prognostic tool, applicable in a variety of contexts including ICIs alone, in combination with other therapies, or across different treatment lines. Additionally, it’s crucial to recognize the mounting body of evidence that highlights the oncogenic potential of HOXC9, thus positioning it as a key target for tailored therapeutic strategies within LUAD immunotherapy. Further studies are warranted to validate our findings and improve our understanding of the oncogenic mechanisms of HOXC9.

## Supplementary Material

Supplementary Figures

Supplementary Table 1
