# Supplementary material for: HOXC9 characterizes a suppressive tumor immune microenvironment and integration with multiple immune biomarkers predicts response to PD-1 blockade plus chemotherapy in lung adenocarcinoma
Source: Aging (Albany NY). 2024 Mar 5;16(5):4841–61. doi: 10.18632/aging.205637 (PMC10968688; doi:10.18632/aging.205637)
Supplement: Supplementary Figures [file aging-16-205637-s001.pdf]

SUPPLEMENTARY FIGURES

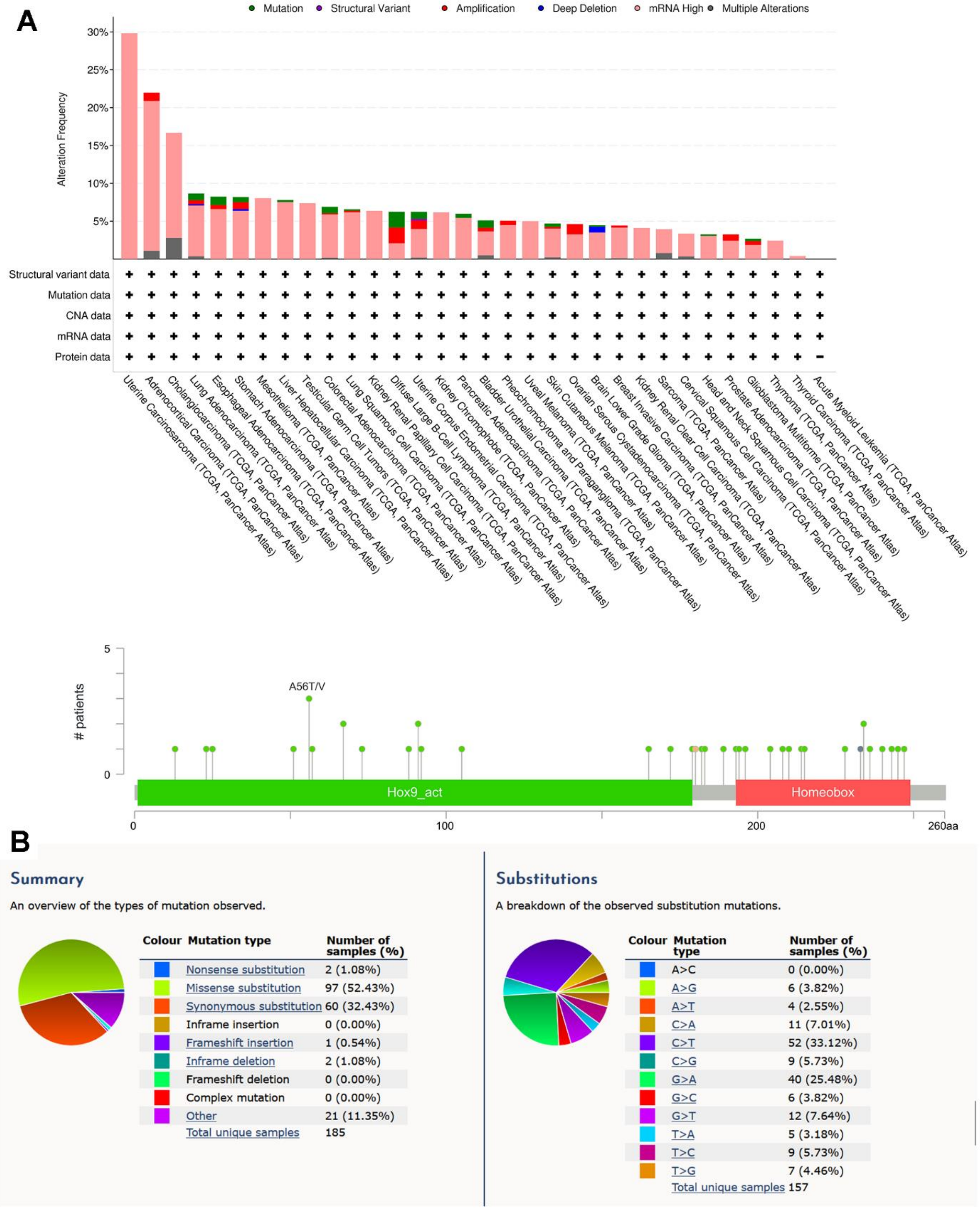

Supplementary Figure 1. HOXC9 genetic alteration and promoter methylation analysis. (**A**) HOXC9 alteration in pan-cancers. (**B**) Types of HOXC9 mutation in pan-cancers. Types and substitution of HOXC9 mutation in pan-cancers.

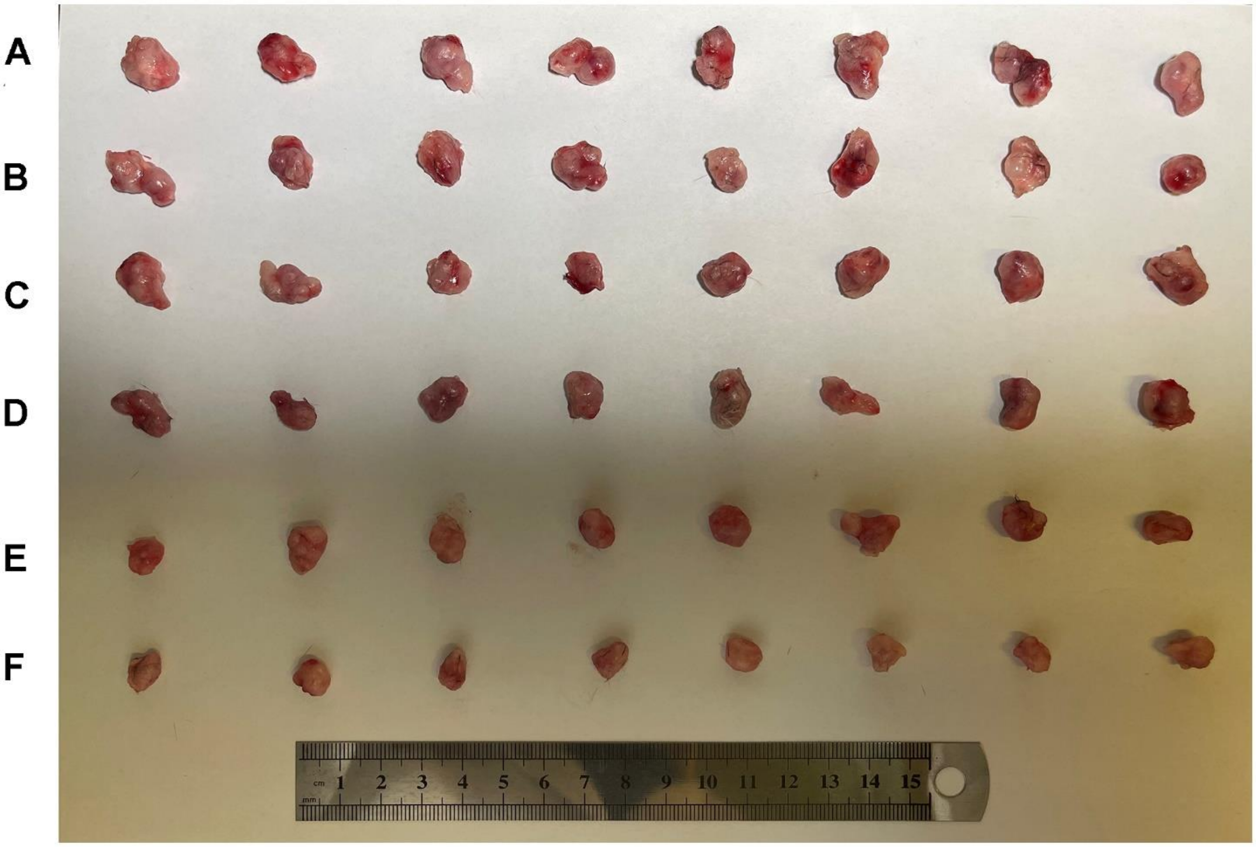

Supplementary Figure 2. Tumor model photograph. (**A**) Control+IgG group. (**B**) Control+anti-PD1 group. (**C**) Over expression of Hoxc9+IgG group. (**D**) Over expression of Hoxc9+anti-PD1 group. (**E**) Knockdown of Hoxc9+IgG group. (**F**) Knockdown of Hoxc9+ anti-PD1 group.
