# Supplementary material for: HOXC9 characterizes a suppressive tumor immune microenvironment and integration with multiple immune biomarkers predicts response to PD-1 blockade plus chemotherapy in lung adenocarcinoma
Source: Aging (Albany NY). 2024 Mar 5;16(5):4841–61. doi: 10.18632/aging.205637 (PMC10968688; doi:10.18632/aging.205637)
Supplement: Supplementary Table 1 [file aging-16-205637-s002.pdf]

SUPPLEMENTARY TABLE

Supplementary Table 1. Baseline characteristics of the included patients.

Characteristics	Patients (n, %)
Age (years)	
<65	19 (61.3)
≥65	12 (38.7)
Gender	
Male	22 (71.0)
Female	9 (29.0)
ECOG performance status	
0	10 (32.3)
1	21 (67.7)
Smoking history	
Non-smoker	11 (35.5)
Smoker	20 (64.5)
PD-L1 TPS	
<1%	11 (35.5)
<49%	15 (48.4)
≥50%	5 (16.1)
Response to treatment	
Complete response	1 (3.2)
Partial response	18 (58.1)
Stable disease	10 (32.3)
Progression disease	3 (9.7)
